# Users’ perspectives on the quality of family planning services in Mozambique: a case study

**DOI:** 10.11604/pamj.2022.42.174.26049

**Published:** 2022-07-04

**Authors:** Paulo Pires, Martins Mupueleque, Cynthia Macaringue, David Zakus, Ronald Siemens, Celso Belo

**Affiliations:** 1 Faculty of Health Sciences, Lúrio University, Bairro de Marrere, Rua, Nr. 4250, km 2.3, CP 364, Nampula, Mozambique,; 2 Faculty of Agriculture, University Mussa Bin Bique, Nampula, Mozambique,; 3 Faculty of Health Sciences, Lúrio University, Nampula, Mozambique,; 4 Faculty of Health Sciences, Ryerson University, Saskatoon, Canada,; 5 Faculty of Medicine, University of Saskatchewan, Saskatoon, Canada

**Keywords:** Contraception, family planning, Mozambique, primary health care, sexual reproductive health

## Abstract

**Introduction:**

user satisfaction assessment has been increasingly important in ensuring health care quality and guidance in the health sector. Africa is the region in the world with the lowest prevalence of contraception use. Universal access to family planning has one of the highest rates of benefits for cost among strategic options for development. Family planning can reduce mortality associated with pregnancy, childbirth, and postpartum, and family planning consultations are available in primary health care in Mozambique since 1980, with services delivered at all different levels of the national public health system. In 2017 this country had a high maternal mortality rate and one of the known causes was the low use of family planning. Among other causes for low utilization is the bias introduced by health professionals in the prescription of contraceptives and service deficiencies. We intended to assess the users’ opinion about family planning visit quality at the Marrere Health Center, to understand how we might consequently increase the use and quality of these services. This study was designed as a planned intermediary evaluation, as part of a larger implementation research project, aiming to reduce maternal and newborn mortality rates in Natikiri neighbourhood, in Nampula peri-urban area.

**Methods:**

we carried out a descriptive cross- sectional quantitative case study, where the target population was users of family planning services at a local health center. A random sample of 137 individuals answered a survey consisting of satisfaction, and we consulted family planning service statistical indicators. Users were considered satisfied if they answered “satisfied” or “very satisfied” on questions. Frequencies were calculated with statistical package for the social sciences (SPSS) 22.0 with a confidence interval of 95% and a 5% margin of error. The study protocol was approved by Lúrio University and the University of Saskatchewan (Canada) Ethics Committees.

**Results:**

almost all respondents (93%), were adult women with low levels of education. They stated, in general (88%), they were satisfied with the quality of care in the family planning consultation. There was minor participation of men in family planning. However, health professionals were found to not generally follow a patient-centered service protocol, with weaknesses in communication and information sharing, more commonly with adolescent clients.

**Conclusion:**

most family planning visits users were adult women, satisfied with the service provided. Successful changes in family planning practice require broad support from political, religious and community sectors. Additionally, proper technical and professional training of health professionals is necessary to achieve a positive impact on individuals, families, communities, and government. Family planning promotion in primary health care is recommended as an important strategy for achieving universal health coverage, protecting the lives of women, and promoting the country's development.

## Introduction

In most national healthcare systems, quality assessment is limited to the perspective of caregivers or health policymakers; and many studies are still designed according to these criteria. User´s opinions have not yet universally received full legitimacy to evaluate the quality of care. However, user satisfaction assessment has been increasingly important in ensuring health care quality and guidance in the health sector, with both services and policy. Africa is the region in the world with the lowest prevalence of contraception use (33% of women of sexually active age) [1]. Mozambique, a country of scarce resources in sub-Saharan Africa, has a high fertility rate (5.2 children per woman), a widening of the pyramid age base (53% of the population is less than 17-years old), and an increasing population that threatens the country's development [2]. Universal access to family planning (FP) has one of the highest rates of benefits for cost among all the various strategic options for development [3]. One of the goals of the strategic guidelines of the World Health Organization (WHO) on FP, is to maximize the impact of explicit and tacit knowledge, including research, through its dissemination and application) [4]. In 2017 Mozambique had a high maternal mortality rate (451.6 maternal deaths per 100,000 live births) and one of the known causes was the low use of FP, especially in rural areas, where most of the population (66.6%) lives.

Among other causes for low utilization is the bias introduced by health professionals (HPs) in the prescription of contraceptives, due to prejudice and insufficient training, as has been reported in other African countries [5]. Family planning can reduce mortality associated with pregnancy, childbirth, and postpartum. Effective use of contraception reduces mortality, by enabling women to delay first pregnancies, space births more widely, and reduce the number of unwanted pregnancies, which sometimes end in clandestine induced abortion [6]. These facts led the Mozambican Government to place FP as a priority in the Strategic health plan 2014 - 2019 [7]. Family planning consultations (FPCs) are part of the women and children's health program (WCH), available in primary health care (PHC), and provided in health centers (HC). Family planning services began in 1977 as an intervention within the WCH protection program, and then were established nationwide in 1980, with services delivered at all different levels of the national public health system [8]. The use of modern contraceptives was estimated in a recent study in Natikiri, a district on the outskirts of the city of Nampula, the capital of the province with the same name, at 47% for adults and 18% for adolescents [9]. Among the causes that lead to such a low use of contraception are PHC services deficiencies, as reported in a baseline study conducted by our team at the very beginning of the research. The baseline study showed a low level of knowledge of sexual and reproductive health (SRH) and FP [10]. So we intended to assess the users’ opinion about FP visits quality at the Marrere Health Center (MHC), to further understand how we might consequently increase the use and quality of the FP services.

## Methods

**Study design:** we deployed a descriptive cross-sectional study.

**Study setting:** surveys applied from 28^th^ June to 3^rd^ July at the family planning services at MHC in Natikiri administrative neighbourhood, Nampula City, Northern Mozambique.

**Participants:** a sample of 137 users of one FP visit in MHC. Non-probability sampling methods were used (i.e, opportunity sampling and convenience sampling). All participants signed an informed consent term, including a separate agreement for those under 18 years of age, and answered verbally in *Macua* (local language) or Portuguese as they preferred. The survey took about 20 minutes on average to complete.

**Variables:** independent variables were age in years, gender, the community of residence, educational level, and obstetric history (i.e, number of pregnancies, place of delivery, number of spontaneous and provoked interruptions of pregnancy, and date of first ante-natal consultation). Dependent variables focused on the assessment of the principles of good care, including communication with the patient, privacy and confidentiality, information about FP, and general satisfaction. Further, open questions explored positive and negative aspects of service delivery. The survey was designed based on a five-point Likert scale (i.e, always, most often, sometimes, rarely, never).

**Data sources:** this study was conducted by a team from the Faculty of Health Sciences (FHS) of Lúrio University (UniLúrio) in partnership with the University of Saskatchewan in Canada, as part of the implementation research project alert community for a prepared hospital care continuum (ACPH), which spanned from 2016-2020. It took place during the 5th semester of ACPH project, and after conducting two years of health education interventions on SRH and FP with the local population. In addition, prior to conducting this study, we trained the health professionals (HPs) in humanized care and family-friendly visits, as well as introduced various technological improvements in the health unit (HU) (e.g. obstetric ultrasound and a new operating room for Cesarean sections). Participants were asked to take part in a closed survey, including three open questions, previously tested, and validated. Surveys were administered by students from UniLúrio´ s FHS Research Group, professionally trained, unknown by the subjects, and after signing an ethical and scientific commitment form.

**Bias:** participants´ potential bias was addressed by pretesting the questionnaire in a local language, applying it by an unknown research assistant, and assuring a private setting without any HP in total confidentiality. Data collection' potential bias was addressed, evaluating the questionnaires´ quality of filling by two different research assistants and a principal investigator.

**Study size**: the sample size was defined considering a margin of error of 5% in the evaluation of the studied parameters, for the monthly population average using those services (161 consultations per month over the first half of 2019 which had 966 users). The level of precision desired for each indicator was based on the amplitude of the 95% confidence interval. The formula used to calculate the sample size was


$$n=\frac{N\times Z^{2}\times P\times (1-P)}{Z^{2}\times P\times (1-P)+E^{2}\times (N-1)}$$

(n = sample size, N = study population size, Z = degree of confidence (0.95), p = constant equal to 0.5, E = margin of error 0.05). The number calculated for the sample was 114. A 10% margin was added for surveys with a lack of information and 10% for surveys with incorrect information, yielding a proposed study group of 137 participants.

**Quantitative variables:** the level of precision desired for each indicator was based on the amplitude of the 95% confidence interval.

**Statistical methods:** the data were introduced in a database by the same students supervised by a statistics Professor [11]; then proceeded to the descriptive statistical treatment of data using the Statistical Package for the Social Sciences (SPSS®) 22.0, having calculated absolute and relative frequencies, mean and standard deviation. No subgroups were considered.

**Ethics:** the study complied with all the recommendations of the Declaration of Helsinki (2013) and was reviewed and approved by the Institutional Committee on Bioethics for Health at Lúrio University (02/CBISUL/16), as well as by the Behavioral Research Ethics Board of the University of Saskatchewan (15-112).

## Results

We surveyed 141 FP visit users, all belonging to the ethnic-language group *Emacua*, 131 (92.9%) were female with a mean age of 27.7 years (minimum 14, maximum 68). Of these, 14 (9.9%) were adolescents, one male, and 13 (93%) female, between 14 and 18 years old (average age of 16.6 years). Of the 139 households evaluated, 107 (77%) come from Natikiri, and the rest from urban Nampula. Their attained school level is detailed in Table 1. Evaluating the number of pregnancies (user's own figure or that of their companion), we found an average of 3.7 pregnancies per woman (minimum 0 and maximum 19). In the adolescent group, the number of pregnancies varies between 0 and 3 (average 0.8). Of 141 evaluated users, 21 (14.8%) never had a pregnancy; of 120 who had been pregnant, 52 were unaware of the date of the first antenatal consultation (ANC) and 69 (57%) were able to inform. On average, they attended the 1^st^ ANC in week 20 (2^nd^ trimester); 22 (32%) performed it during the 1^st^ trimester. Regarding the number of hospital deliveries, we found an average of 2.9 per user (minimum 0, maximum 10), and the occurrence of 67 home births (an average of 0.49 per user, minimum 0 and maximum 11). In the adolescent group, 8 (62%) had 9 pregnancies (average 1.1) and they all delivered at hospital maternity, with no home deliveries being reported. Spontaneous pregnancy interruption (miscarriage) was reported in 30 cases (an average of 0.22 per user, minimum 0, and maximum 3) and voluntary interruption in 11 cases (an average of 0.08 per user, minimum 0, and maximum 2). In the adolescent group, spontaneous pregnancy interruptions were not reported, but one case with two voluntary interruptions (an average of 0.2 per user, minimum 0, and maximum 2). The assessment of principles of good care is detailed in Table 2. We highlight the following results: the users are greeted and they are offered a seat (always 65%, most of the time 11.6%); the HP asks their name (always 75.5%, for adolescents 69.2%); users feel welcome at the consultation (always 60.7%, for teenagers 57%); they are motivated to return to the consultation (always 54.7%, for adolescents 64%).

**Table 1:** educational level of users of the family planning consultation

	Total	
Educational level	No	%
Illiterate and incomplete primary	59	41.8
Complete primary	69	48.9
Complete secondary	13	9.2
Higher or technical-professional	0	0
Total	141	100

No: number; %: percentage

**Table 2:** level of users' satisfaction at family planning consultation

Group		Teens					Adults				
No.	Question / number of respondents (14 adolescents, 141 in total), frequency (%)	Always	Most times	Sometimes	Few times	Never	Always	Most times	Sometimes	Few times	Never
	**Principles of good care**										
1	It was greeted and offered a seat	9 (64)	0 (0)	2 (14)	1 (7)	2 (14)	70 (63)	16 (14)	12 (11)	4 (4)	9 (8)
2	The health professional introduced himself	4 (29)	1 (7)	1 (7)	2 (14)	6 (43)	29 (26)	8 (7)	6 (6)	6 (6)	61 (56)
3	The health professional asked your name	9 (69)	2 (15)	1 (8)	0 (0)	1 (8)	86 (78)	8 (7)	6 (5)	6 (5)	5 (5)
4	The health professional explained what would happen during the consultation	6 (43)	1 (7)	0 (0)	2 (14)	5 (36)	45 (41)	14 (13)	15 (14)	8 (7)	29 (26)
5	At the end of the visit, the health professional asked whether she had any doubts	6 (43)	0 (0)	0 (0)	2 (14)	6 (43)	42 (38)	17 (15)	3 (3)	8 (7)	41 (37)
6	The health professional summarized the most important information at the end of the consultation	6 (43)	0 (0)	1 (7)	2 (14)	5 (36)	52 (47)	17 (15)	13 (12)	11 (10)	18 (16)
7	Feels motivated to return to the next appointment or before if you have questions	9 (64)	2 (14)	0 (0)	2 (14)	1 (7)	59 (54)	19 (17)	13 (12)	7 (6)	12 (11)
8	Felt welcome to the consultation	8 (57)	4 (29)	0 (0)	2 (14)	0 (0)	65 (59)	25 (23)	10 (9)	6 (5)	5 (5)
9	The health professional encouraged her to ask questions	7 (50)	1 (7)	2 (14)	0 (0)	4 (29)	42 (38)	15 (14)	5 (5)	15 (14)	34 (31)
10	If there was any concern, the health professional clarified amicably using simple and clear language	9 (64)	1 (7)	1 (7)	1 (7)	2 (14)	54 (49)	15 (14)	15 (14)	11 (10)	16 (14)
11	During the consultation, the health professional clearly clarified issues related to her health status	5 (36)	2 (14)	2 (14)	1 (7)	4 (29)	51 (46)	17 (16)	10 (9)	14 (13)	18 (16)
12	Before starting the consultation, the health professional asked what her expectations were	4 (29)	1 (7)	1 (7)	1 (7)	7 (50)	33 (30)	13 (12)	10 (9)	10 (9)	45 (41)
13	Before performing the physical examination or any intervention the health professional explained what he would do	8 (57)	0 (0)	0 (0)	2 (14)	4 (29)	53 (48)	8 (7)	11 (10)	14 (13)	23 (23)
14	The health professional encouraged the participation of her husband/partner/during the consultation	1 (7)	2 (14)	1 (7)	0 (0)	10 (71)	35 (32)	6 (6)	7 (6)	10 (9)	52 (47)
1 5	A private place was provided for examination and counselling	10 (71)	0 (0)	1 (7)	0 (0)	3 (21)	67 (60)	15 (14)	11 (10)	12 (11)	6 (5)
1 6	The provider did something to make sure no one was listening, when discussing sensitive issues	10 (71)	0 (0)	1 (7)	1 (7)	2 (14)	65 (59)	14 (13)	9 (8)	13 (12)	10 (9)
17	Before talking to your partner or family about sensitive information, the healthcare professional obtained your consent	8 (57)	0 (0)	1 (7)	0 (0)	5 (36)	43 (39)	12 (11)	12 (11)	13 (12)	41 (37)
1 8	At the end of the visit you were assured that your personal information will not be discussed with other people	5 (36)	1 (7)	1 (7)	0 (0)	7 (50)	47 (42)	3 (3)	12 (11)	8 (7)	41 (37)
19	Was she informed about FP advantages?	9 (64)	0 (0)	0 (0)	1 (7)	4 (29)	66 (60)	9 (8)	6 (6)	9 (8)	20 (18)
2 0	Was she informed about 3 contraceptive methods?	10 (71)	0 (0)	0 (0)	2 (14)	2 (14)	59 (54)	9 (8)	12 (11)	8 (7)	22 (20)
2 1	Have you been informed about contraceptives side effects?	4 (29)	1 (7)	2 (14)	1 (7)	6 (43)	37 (34)	7 (6)	7 (6)	15 (14)	44 (49)

Other responses are less favorable: the HP does not present himself (never 51.8%, 43% for adolescents); the HP explains what will happen during the visit (always 43.6%); clearly clarifies issues related to health status (always 46%, for adolescents 36%); asks if they have any questions (never 40%, for teens 43%) or summarizes the most important information at the end of the consultation (always 48.6%, for teenagers 43%). Concerning communication with the users, HPs do encourage little participation of the husband, partner, or companion (never 49.6%, for teens 71%); clarify doubts cordially using simple and clear language (always 51.4%, for adolescents 64%); encourage them to ask questions (always 39.3%, for teenagers 50%). “Asking about users´ expectations” never occurred with 41.4% (50% for teens) and the explanation of physical examination was reported by a small majority (always 50%, for teens 57%). Appropriate privacy during the appointments was mentioned by most users (always 61.4%) as well as the absence of third parties (always 62.1%). Confidentiality information was reported by a minority (always 42.1%, for adolescents 36%) as well as consent for family information about health issues (always 38.6%, for adolescents 57%). Most users were informed about FP advantages (always 61.9%) and about three different types of contraceptives (always 56.1%, for adolescents 71%), but about contraceptive side effects, a significant number (never 39.6%, adolescents 43%) did not receive information. The overall evaluation of users´ satisfaction in the FP visits shows the majority are satisfied (Table 3).

**Table 3:** monthly statistical summary of family planning consultation service at Marrere Health Center, May 2019

Study group	Total		Teens	
Level	No	%	No	%
Very satisfied	32	24.3	2	14
Pleased	89	63.6	11	79
Not very satisfied	16	11.4	1	7
Dissatisfied	1	0.7	0	0
Total	138	100	14	100

No: number; %: percentage

Answering the open question about what they liked about the service, 130 women (90.9%) liked it in general (good service, information, provision of contraceptives, professional empathy, counseling, speed), 4 (2.8%) do not comment and 9 (6.3%) did not like it. Answering the open question about what they did not like about the service, 82 women (57.7%) liked everything without pointing out any negative points; 18 (12.7%) did not comment, and 42 (29.6%) reported several problems: no clarification of doubts (8.5%), poor communication, lack of privacy, poor quality service (each respectively 2.1%), illegal charges, consultation interruptions to speak on the phone (each respectively 1.4%). Answering the open question about what they would change to make the service better, 52 women (36.6%) pointed out several issues, referring to the need to improve service quality (10.6%) and communication (8.5%), speed in attendance (6.3%), contraceptives availability (2.8%), eliminating illegal charges, facilitating a change of methods, not communicating on the phone during the consultation (each 2.1% respectively, improving privacy (1.4%); 62 (43.7%) users would do nothing and 28 (19.7%) do not know how to answer.

## Discussion

Most users of FP visits are adult women with low school level, and do not bring their partner to the consultation, confirming the FP service monthly statistical summary relating to May 2019 Table 4, documenting activities carried out in the FP consultation. Our study group was generally satisfied with the quality of care in the FP consultations service, though the HPs did not always proceed according to the rules of good care and the FP protocol of the Ministry of Health (MOH). These FP service users live predominantly in rural areas and 85% had been pregnant (an average of 4 pregnancies per woman, below the Mozambican fertility rate, considering that the group is on average 28 years old). Of these 57% are able to inform the date of the first ANC (on average in the 20^th^ week, 2^nd^ trimester), compared with 32% having performed it in the first trimester, according to other studies carried out in developing countries [11]. The average number of 2.9 hospital deliveries per woman is under the pregnancy rate, resulting from the persistence of home births, when delivery is supported by a traditional birth attendant. Our results also showed that there was a little participation by men during the appointments. We deemed this to be less than optimal as FP services and the discussions involved should also include the husbands.

**Table 4:** answers to the survey on family planning visits quality assessment, in the opinion of users

	Group		
No	Service (n)	Total 1^st^ consultation	Total following visits
1	SRH/FP	183	285
		**Total users**	
2	Total consultations	468	
3	STIs	1	
4	Breast examination	161	
5	CCU screening	87	
6	Via + test	9	
7	Screening syphilis	180	
8	Syphilis +	5	
9	Syphilis incidence (%)	2.8	
10	HIV screening	468	
11	HIV +	62	
12	HIV incidence (%)	13.2	
13	Partners present	5	
14	Pill	53	89
15	Injectable	107	273
16	IUD	3	2
17	Implant	21	21

The HPs were found to have shortcomings in information and communication with patients, for example the general lack of self-presentation or the lack of information on contraceptives, more pronounced in the group of teenagers. However, international human rights law requires HUs, services and contraceptives to be accessible to all without discrimination, including physical and economic access as well as access to information [12]. Health professionals, though, generally comply with MOH guidelines regarding privacy and confidentiality requirements, but less so with adolescents, demonstrating the need to deepen their technical and social preparation. These findings are like those in another study in Burkina Faso [13]. In general, users reported complaints, leading to recommendations to improve the service: better quality of care (enhanced communication with HP, including the HP not to talk on the phone during the consultation), reduce waiting delay, increase contraceptives availability, end illicit charges, facilitate the change of methods, and enhance privacy.

It has been demonstrated that successful changes in the practice of FP need a broad support, from political commitment beyond the health sector, from notable champions and partner collaboration, religious and community leaders, community provision of services, establishment of effective strategies and systems, and with adequate technical preparation of HPs [14]. In developing countries, FP programs must include strategic interventions to reduce unmet needs for contraception and unwanted early pregnancies in adolescents [15]. It is also recommended to have patients at the center of health care strategies to ensure the effectiveness and efficiency of PHC [16]. For this to happen they must somehow be part of the decision-making process. As limitations of this study, we point the location where the surveys were administered (inside MHC). Hence, we believe that some answers might have been influenced by this environment. The application of a likert rating scale may have been confusing to participants, due to the fact that our sample had basic education and perhaps had difficulties on abstract conceptualization, especially Macua speakers in the interpretation of terms like “always” and “often”, “sometimes” and “rarely”. We should also consider that services evaluation by means of production statistics, being a major activity for the planning and management of health services, still lacks an organic and integrated approach, adapted to the HPs skills [17].

## Conclusion

The low use of contraceptive methods increases fertility rates in Mozambican women, explaining the African demographic explosion. Family Planing services are central to all maternal health programs, and we found that most FP service users at MHC were satisfied with the consultation, though with some incisive criticism and suggestions. HP's were generally practicing according to the MOH privacy and confidentiality protocol, but there are still important deficiencies in good care principles, especially with communication and information with users, particularly regarding contraceptive side effects and enabling access to contraceptives, which are more pronounced when it comes to the adolescent group, and one of the reasons for poor FP adherence. Reinforcement of HP's training for FP consultation is recommended, with the objective of promoting FP in PHC and protecting women and families´ health, including the reduction in adolescent pregnancies. Overall, the provision of such FP services is an important strategy to achieve universal health coverage and promote the country's development.

### What is known about this topic

- Maternal and child mortality are high in Mozambique and an important cause is low use of contraceptives;
- Family planning practice depends on health services quality and professional's performance.

### What this study adds

- Family planning services users in Natikiri are mostly satisfied with the quality of care;
- Health professionals training should target adolescent communication and sexual and reproductive health education.
